# The clinicopathological landscape of thyroid cancer in South Africa—A multi‐institutional review

**DOI:** 10.1002/wjs.12353

**Published:** 2024-09-22

**Authors:** Wilhelmina Conradie, Thifhelimbilu Luvhengo, Jeanne Adele Lübbe, Amir Afrogheh, Aneldi Bestbier, Mirza Bhuiyan, Ifongo Bombil, Sharon Raye Čačala, Lydia Cairncross, Chanel Changfoot, Jenny Edge, Brandon S. Jackson, Hansjörg S. Jehle, Lusanda Jonas, Mpoyi Ruphin Lukusa, Malose Makgoka, Lindi Martin, Daniel Nel, Mohamed Quraish Patel, Nosisa Thabile Sishuba, Rubina Razack, Karin Baatjes

**Affiliations:** ^1^ Department of Surgery Tygerberg Hospital University of Stellenbosch Cape Town South Africa; ^2^ Department of Surgery University of Witwatersrand Johannesburg South Africa; ^3^ Department of Oral and Maxillofacial Pathology University of the Western Cape and National Health Laboratory Service (NHLS) Cape Town South Africa; ^4^ Port Elizabeth Hospital Complex Walter Sisulu University Gqeberha South Africa; ^5^ Department of General Surgery Mankweng Academic Hospital University of Limpopo Pretoria South Africa; ^6^ Department of Surgery Groote Schuur Hospital University of Cape Town Cape Town South Africa; ^7^ University of Witwatersrand Charlotte Maxeke Hospital Johannesburg South Africa; ^8^ Kalafong Provincial Tertiary Hospital University of Pretoria Pretoria South Africa; ^9^ Pietersburg Hospital University of Limpopo Polokwane South Africa; ^10^ Grey's Hospital University of KwaZulu‐Natal Pietermaritzburg South Africa; ^11^ Department of General Surgery Steve Biko Academic Hospital Pretoria South Africa; ^12^ Division of General Surgery Department of Surgery University of Cape Town Cape Town South Africa; ^13^ National Health Laboratory Service Division of Anatomical Pathology Faculty of Medicine and Health Sciences University of Stellenbosch Cape Town South Africa; ^14^ Department Surgical Sciences University of Stellenbosch Cape Town South Africa

**Keywords:** Africa, low‐to‐middle income country, South Africa, thyroid, thyroid cancer

## Abstract

**Background:**

In South Africa (SA), data on the incidence of thyroid cancer is limited. Papillary thyroid carcinoma is by far the most common malignancy in developed countries; however, a preponderance of follicular thyroid cancer in developing countries, despite iodized salt, has been observed. The aim of this study was to describe the national landscape of thyroid cancer in SA with reference to pathological subtypes, surgical outcomes, and treatments offered.

**Methods:**

A multi‐institutional retrospective review of thyroid cancer patients operated on between January 2015 and December 2019 was performed. Public hospitals with associated academic institutions were included. Data were collected from theater registers, pathology, and radiology records. Statistical analysis was done to determine intergroup significance.

**Results:**

A total of 464 thyroid cancer cases from 13 centers across five SA provinces were identified. Most patients presented with a mass (67%). Ultrasound was performed in 82% of patients, and 16.3% underwent surgery without pre‐operative cytology. Of the histologically confirmed thyroid cancers, 61.8% were papillary and 22.1% follicular thyroid cancer. There was a significant association between subtype and geographical area, and T‐stage and operation performed. Surgical complication rates included hematoma in 1.8%, post‐operative hypocalcemia in 28.7%, and recurrent laryngeal nerve injury in 3.5%.

**Conclusion:**

This first national review describes the landscape of thyroid cancer in SA, revealing considerable differences compared to international studies. It provides valuable insight into the unique South African experience with this disease. In addition, this study serves as an impetus towards a prospective national registry with real‐world data informing contextualized guidelines.

## 1 INTRODUCTION

Thyroid cancer is the most common endocrine malignancy globally and is increasing in prevalence.^1^, ^2^ In South Africa (SA), a country marked by a disparate health system, the data on the incidence of thyroid cancer is limited. Single‐center studies from SA revealed a contrasting prevalence compared to high‐income countries, where papillary thyroid carcinoma (PTC) accounts for 80% of cases and follicular thyroid carcinoma (FTC) is more common in iodine‐deficient areas.^3^, ^4^, ^5^ Despite iodized table salt already introduced in SA in 1982, the incidence of FTC remains higher than in developed countries.^4^ In the study conducted by Robertson and colleagues, PTC was the most common (60.6%), followed by FTC (38.9%), with poorly differentiated thyroid cancer (PDTC) being the rarest (0.5%).^5^ Of the 5.7% of patients with multinodular goiter found to have incidental thyroid cancer at thyroidectomy, Bombil et al. determined that all 70 cases were PTC, of which 75% was the follicular variant of PTC.^6^

Diagnosing thyroid cancer involves evaluating the nodule risk of malignancy based on clinical, sonographic, and cytological assessment, with molecular tests, when available.^7^, ^8^ Management depends on the subtype and stage and requires a multidisciplinary approach with surgery as the mainstay of treatment. Adjuvant treatment in high‐risk, well‐differentiated thyroid cancer (WDTC) includes radioactive iodine (RAI) and suppression of thyroid stimulating hormone (TSH).^9^ Medullary thyroid cancer (MTC) has a higher risk of lymph node metastases and is, therefore, treated with sat least a total thyroidectomy and central lymph node dissection.^10^ Thyroidectomy is not routinely performed in patients with primary thyroid lymphoma or anaplastic thyroid cancer (ATC).^7^, ^11^

The safety of thyroid surgery increases with the surgeon's experience.^12^ Bleeding and recurrent laryngeal nerve (RLN) injury occur in 0.1%–3% and 2.3% of patients, respectively,^13^ and are more common in reoperations and surgery for malignancy.^14^ Complication rates in SA are poorly documented, and thyroidectomies are not always performed in high‐volume centers.

Thyroid carcinoma guidelines are developed by and for high‐income countries and are not universally applicable to an economically, culturally, and geographically diverse country such as SA.^15^ This study by the Thyroid Cancer Group of South Africa (TCGSA) aimed to describe the national landscape of thyroid cancer in the public sector in SA with reference to pathological subtypes, surgical outcomes, and treatments offered.

## 2 MATERIALS AND METHODS

A multi‐institutional retrospective review of thyroid cancer patients operated on between January 2015 and December 2019 was performed, excluding 2020 due to possible bias from the COVID‐19 pandemic. Twenty‐six institutions initially agreed to partake in this study, of which 13 (all public hospitals with associated academic institutions) were eventually included. Reasons for losing initially recruited institutions were: a delay in granting of ethics approval by the local university, lack of time to obtain retrospective data, loss of communication with the core investigators, lack of administrative support, and delegating the task to a junior colleague. Patients <12 years were excluded. Study data were collected and managed using Research Electronic Data Capture (REDCap) hosted at Stellenbosch University. REDCap is a secure, web‐based software platform that supports data capture for research studies.^16^ Data was collected from theater registers, pathology reports, and radiology records and included demographic information, investigations and results, indications and types of surgical procedures performed, postoperative outcomes, and TSH suppression and RAI treatment.

Means with standard deviations or medians and interquartile ranges for continuous variables and counts and percentages for categorical variables were computed. Differences between independent groups (provincial location, operation type, and histological subtype) for continuous variables were evaluated using Student's *t*‐tests or Mann‐Whitney U‐tests. The Chi‐squared or Fisher's exact tests were used to test the significance of associations between categorical variables. The waiting time for surgery was defined as the time between cytological diagnosis and surgery. Statistical significance was set at *p* < 0.05. This study was approved by the Health Research Ethics Committee of the University of Stellenbosch.

## 3 RESULTS

**FIGURE 1:**
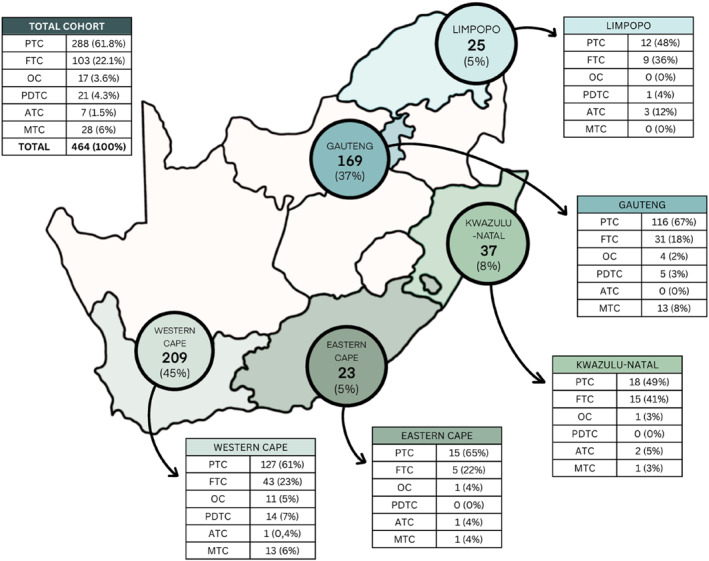
South African provinces that contributed to the national thyroid cancer audit, with a geographic representation of the pathological subtypes of thyroid cancer. ATC, anaplastic thyroid carcinoma; FTC, follicular thyroid carcinoma; MTC, medullary thyroid carcinoma; OC, oncocytic thyroid carcinoma; PDTC, poorly differentiated thyroid carcinoma; PTC, Papillary thyroid carcinoma.

The TCGSA collectively entered 464 thyroid cancer cases from 13 centers across five SA provinces into a REDCap database (Figure 1). Data obtained from some facilities were either lacking or incomplete. Of 464, 458 records included sex, of which 371 (79.6%) were female and 87 (18.7%) were male. Demographics are described in Table 1.

**TABLE 1:** Demographics of thyroid cancer patients in South Africa.

Sex *N* = 458	Female	371 (81.0%)
	Male	87 (19.0%)
Age *N* = 409	Years	13–90 (mean 49.5, SD 15)
Functionality *N* = 450	Euthyroid	377 (83.8%)
	Hypothyroid	31 (6.9%)
	Hyperthyroid	28 (6.2%)
	Subclinical hyperthyroidism	8 (1.8%)
	Subclinical hypothyroidism	6 (1.3%)
Thyroid medication *N* = 463	Thyroxine	64 (13.8%)
	Carbimazole	14 (3.0%)

The presenting symptoms included thyroid mass in 67.2% (272) patients, metastatic disease in 4.0% (16) of patients, and incidental diagnosis in 3.7% (15) (Table 2).

**TABLE 2:** Presenting symptoms, clinical findings and diagnostic work‐up of operated South African thyroid cancer patients.

Presenting symptoms in 405/464 (% of total reported)	405 (100%)
Mass	272 (67.2%)
Compression	66 (16.3%)
Hoarseness	18 (4.4%)
Incidental	15 (3.7%)
Metastatic disease (thyroid primary)	16 (4.0%)
Metastatic screening for extrathyroidal malignancy	4 (1.0%)
Lymph adenopathy	7 (1.7%)
Functional abnormality	7 (1.7%)
Clinical morphology in 375/464 (% of total reported)	375 (100%)
Diffusely enlarged	75 (20.0%)
Multinodular goiter bilateral	59 (15.7%)
Multinodular goiter unilateral	71 (18.9%)
Solitary nodule	158 (42.1%)
Normal	12 (3.2%)
Cytology per TBSRTC category in 360/430 (% of total reported)	360 (100%)
I	32 (8.9%)
II	30 (8.3%)
III	29 (8.1%)
IV	69 (19.2%)
V	40 (11.1%)
VI	116 (32.2%)
Not reported according to TBSRTC	44 (12.2%)

Abbreviation: TBSRTC, The Bethesda System for Reporting Thyroid Cytology.^17^

Ultrasound was performed in 82.2% (351/427) of patients, with only 77.8% (332) reporting more detail. The American College of Radiology Thyroid Imaging Reporting and Data System (ACR TI‐RADS) classification was reported in only 19.6% (65/332) cases. Data on the maximum dimension of the nodule were recorded in 161 patients. Thirty‐five patients had a nodule <19 mm (21.7%), 72 (44.7%) between 20 and 40 mm, and 54 (33.5%) bigger than 41 mm. The median size was 33 mm (IQR 20.7–47.2) (Table 2).

Pre‐operative cytology was performed in 83.8% (360/430) of patients (Table 1). Cytology was reported according to The Bethesda System for Reporting Thyroid Cytology in 87.8% (316) of cases, with 8.3% (30) of thyroid cancers categorized as benign (Bethesda II). The fine needle aspiration biopsy (FNAB) result was indeterminate in 38.3% (138), comprising atypia of undetermined significance (Bethesda III) in 8% (29), follicular neoplasm (Bethesda IV) in 19.2% (69) and suspicious for malignancy (Bethesda V) in 11.1% (40) of patients. In 32.2% (116) of patients, malignant cytology (Bethesda VI) was reported.

Computerized tomography (CT) scans were performed in 27.8% (117/421). Indications for CT scan included bulky neck disease (26.5%, 31), concerning symptomatology (17.9%, 21), retrosternal extension (17.1%, 20), thyroid cancer staging (23.9%, 28) and other (14.5%, 17). Iodine (I‐123) scintigraphy was performed in 56.5% (122/216) of patients.

The nodal and metastatic staging was documented in only 38.8% (180) and 41.4% (192) of patients, respectively, making AJCC stage calculation for all cases impossible. Twenty‐three percent (93) of patients had T1 disease (<2 cm), 20.2% (82) T2 disease (2–4 cm), 48.1% (195) T3 disease (>4 cm or gross extrathyroidal extension invading the infrahyoid muscles) and 7.0% (35) T4 disease (invading surrounding neck structures). Metastases were noted in 38 patients, of which bone and pulmonary involvement were noted in 47.4% (18) and 42.1% (16).

Of the 464 histologically confirmed thyroid cancers, 61.8% (288) were PTC and 22.1% (103) were FTC. The follicular variant of PTC (FVPTC) constituted 66.7% (8/12) of the PTC from Limpopo province, 55.6% (10/18) from Kwazulu‐Natal, and 64.7% (75/116) from Gauteng province.

Six percent (6.6%, 19) of tumors were smaller than 1 cm (microPTC). BRAF mutations were tested in 29.4% (121/411) of tumors and were positive in 18.2% (22/121). Poorly differentiated thyroid carcinoma was found in 4.3% (21) of cases. Sixty‐six (66.6%, 14) of the PDTC were from the Western Cape, and 92.9% (20) of those presenting with T3 and T4 tumors. Six percent (27) of thyroid cancers were MTC, of which 20% (5) had metastatic disease. One and a half percent (1.5%, 7) had ATC. There was a significant association between pathological subtype and province, with significantly more ATC diagnosed in Limpopo, FTC in KwaZulu‐Natal and PDTC in the Western Cape (*p* = < 0.001).

The median waiting time for surgery was 106 days (IQR 54.5–190.5). The indication for surgery was “diagnostic” in 37.3% (170) and “proven malignancy” in 36.4% (166) (Table 3).

**TABLE 3:** Indication for surgery in thyroid cancer patients.

Indication for surgery	*N* = 456 (%)
Diagnostic	170 (37.3)
Compression	79 (17.3)
Acute stridor	4 (0.8)
Retrosternal extension	11 (2.4)
Proven malignancy	166 (36.4)
Completion surgery	14 (3.0)
Cosmetic	25 (5.5)
Functional abnormality	7 (1.5)
Surveillance	2 (0.4)

Total thyroidectomy was performed in 52.2% (242/464) of patients. Of these, 58.3% (168) cases had confirmed PTC and 36.9% (38) FTC, while lobectomies were performed in 38.5% (111) of patients with PTC and 56.3% (58) with FTC (Table 4).

**TABLE 4:** Operation by histological subtype in 464 patients.

	PTC (*n* = 288)	FTC (*n* = 103)	Other (*n* = 73)^a^	Total operation type
Total thyroidectomy	168 (58.3%)	38 (36.9%)	36 (49.3%)	242 (52.2%)
Thyroid lobectomy	111 (38.5%)	58 (56.3%)	30 (41.1%)	199 (42.9%)
Completion thyroidectomy	43 (14.9%)	25 (24.3%)	18 (24.7%)	189 (40.7%)
Lymph node dissection	84 (29.2%)	14 (13.6%)	26 (35.6%)	124 (26.7%)

Abbreviations: FTC, follicular thyroid carcinoma; PTC, papillary thyroid carcinoma.

^a^ Other includes oncocytic carcinoma, poorly differentiated thyroid carcinoma, anaplastic thyroid carcinoma and medullary thyroid carcinoma.

Of the 86/351 patients with WDTC with T1 tumors, 44.2% (38) had a total thyroidectomy (Table 5). There was a significant association between the T‐stage of the cancer and the operation performed (*p* < 0.002). Thyroid lobectomy was more commonly performed in T1 tumors, and more total thyroidectomies were performed in T4 tumors. Thyroid lobectomy and total thyroidectomy were performed at similar rates in 351 WDTC cases with T1 and T2 disease, with total thyroidectomy being more common in T3 and T4 tumors (60.0%). Three percent (2.9%, 13) of patients had a sternotomy, and 1.5% (7/453) had a manubrial split. Reoperations were reported in 2.5% (11/432) of patients.

**TABLE 5:** Thyroid operations per T stage in 351 well‐differentiated thyroid cancer cases.

T Stage	Thyroid lobectomy (%)	Total thyroidectomy (%)	Total (%)
T1	48 (55.8)	38 (44.2)	86 (100)
T2	39 (53.4)	34 (46.6)	73 (100)
T3	68 (40)	101 (60.0)	169 (100)
T4	5 (21.7)	18 (78.3)	23 (100)

For blood vessel ligation, the traditional clip‐and‐tie technique was used in 28.7% (99/345) resections, Ligasure® in 24.6% (85/345), and Harmonic Scalpel® in 51.0% (176/345). An additional hemostatic agent was used in 13.5% (47/345) of resections. Twenty‐six percent (111) were performed with intraoperative nerve monitoring (IONM), and 82.5% (340/412) of surgeons left a drain.

The rate of hematoma requiring reoperation was 1.8% (7/387). Postoperative hypocalcemia and post‐operative RLN injury were evident in 28.7% (100/348) and 3.5% (12/343) of cases, respectively. There was no association between operation type and RLN injury (*p* = 0.295) nor hematoma (*p* = 0.624). However, there was a significant association between operation type and hypocalcemia (*p* < 0.001). More patients with total thyroidectomy developed hypocalcemia compared to those who underwent lobectomy. There was also a significant association between T‐staging and RLN injury: significantly more T3 tumors complicated with RLN injury (*p* = 0.005). No associations were found between the T‐stage and the development of a postoperative hematoma (*p* = 0.540) or hypocalcemia (*p* = 0.111).

Adjuvant RAI was administered to 53.4% (189/354) of the cases. Thirty‐one percent (31.5%, 146/463) of patients had TSH suppression, 0.2% (1/463) had chemotherapy, and 0.4% (2/463) had external beam radiotherapy. Twenty‐two percent (22.7%, 105/463) had no adjuvant treatment.

## 4 DISCUSSION

This first national review describes the landscape of thyroid cancer in SA, revealing considerable differences compared to international studies, providing valuable insight into the unique South African experience with this disease.

A clinical tumor was evident in 67.2% of the cases, and only 3.7% were incidentally diagnosed. Most patients (56.8%) presented with tumors larger than 4 cm or gross extrathyroidal extension with invasion of the infrahyoid muscles. This is in stark contrast to high‐income countries (United States and Canada), where symptomatic thyroid cancers are detected in 30% of cases.^18^ The high rate of symptomatic detection in SA may be due to barriers to healthcare access, resulting in long waiting times for diagnostic and therapeutic interventions.^19^

In this study, 82.2% of patients underwent a preoperative ultrasound. However, only 19.6% of cases were reported according to the ACR TI‐RADS classification. On ultrasound, nodule sizes were documented in 35.2% of reports, with 33.5% of the cases reported as being >41 mm. The median size was 33 mm (IQR 20.7–47.2). Developed countries, on the other hand, have reported a median nodule size of <11.4 mm (IQR 2–96).^20^ The discrepancy in size in our cohort may suggest a diagnostic delay. Inconsistent reporting may be related to ultrasound training, experience, and access. Standardization of reporting facilitates decision‐making, and clinician‐performed ultrasound may further improve ultrasound availability and FNAB accuracy. Improving thyroid ultrasound reporting should be a focus for future interventions.

In thyroid cancer patients in SA, cytological evaluation presented many challenges. In our cohort, 16.3% of patients underwent surgery without pre‐operative cytology, of those with FNAB only 32.2% had a Bethesda VI result, and diagnostic surgery was indicated in 35.6% of cases. This preoperative diagnostic rate highlights the limitations of thyroid FNAB and may be improved by ultrasound guidance or molecular testing. In the absence of molecular testing, the follicular neoplasm category necessitates diagnostic surgery. The higher incidence of FTC in this population might also increase the rate of diagnostic surgery. Establishing high accuracy of FNAB technique and cytological interpretation can facilitate surgical decision‐making and prevent unnecessary surgical procedures.

According to the surveillance, epidemiology and end results data, PTC accounts for 84% and FTC 11% of all thyroid cancers,^21^ although FTC is more common in iodine‐deplete regions.^3^ In our population, 22.1% of cases were FTC. The rate of PTC was 61.8%, lower than the prevalence of 84% reported in the United States. We noted a significant association between pathological subtype and geographic location in SA. There was a significantly higher incidence of ATC in Limpopo province (12.0%), FTC in KwaZulu‐Natal (40.5%), and PDTC in the Western Cape (66.6%) (*p* < 0.001). These geographic differences in thyroid cancer in SA might be skewed by the small number of patients and interobserver variability between pathologists. Future studies focusing on thyroid cancer pathology should explore these differences more thoroughly.

Thyroid surgery is safe in experienced hands. However, the associated complications can be life‐threatening. Common complications of thyroidectomy include postoperative bleeding (0.1%–3%), RLN injury (2.3%), and hypocalcemia (0.5%–65%).^12^, ^14^ In our study, bleeding requiring reoperation occurred in 1.8%, RLN injury in 3.5%, and post‐operative hypocalcemia in 28.7%. Malignancy was the only independent risk factor for reoperation: 4.9% versus 3.3% in the American National Surgical Quality Improvement Program database.^22^ Therefore, a higher complication rate in this cohort undergoing thyroid cancer surgery may be expected.

Pre‐operative voice assessment findings were not captured pre‐operatively; thus, it is unclear whether this number relates to only complications or thyroid cancer invasion. The national post‐operative vocal cord palsy rate of 3.5% is similar to international findings.^13^ There was no association between operation and RLN injury (*p* = 0.295), but a significant association between T‐staging and RLN injury, with more T3 tumors complicated with RLN injury (*p* = 0.005). Intraoperative nerve monitoring (IONM) may improve this complication rate and create awareness around functional assessment of the RLN.^23^

Post‐thyroidectomy hypocalcemia is reported within a wide reference range of 0.5%–65%.^14^ In our cohort, 28.7% were reported to have postoperative hypocalcemia. There was a significant association between hypocalcemia and operation type (*p* < 0.001) but no association with the T‐stage (*p* = 0.111). It was not specified if the hypocalcemia was transient or permanent. The rate of hypocalcaemia may be decreased by less extensive surgery such as lobectomy instead of total thyroidectomy, as well as a cautious approach to parathyroid identification and preservation.

Adjuvant RAI is recommended in intermediate‐risk WDTC and high‐risk patients.^7^ In our study, most patients who received RAI had T3 tumors (37% for PTC and 66.7% for FTC). The treatment approach for the remainder of high‐risk patients is unclear from the data collected and requires further exploration.

## 5 LIMITATIONS

This study is limited by participation bias, with the majority of responses from the Western Cape and Gauteng provinces accounting for 45% and 37%, respectively. These provinces have a larger urban and migrant patient population and a differently resourced healthcare system than the rest of SA. This may skew the pathological subtypes observed in this study. Low participation was also found in less‐resourced provinces. No private healthcare sector cases were included in this cohort, which may influence the generalizability of the findings in SA. Data collection and analysis were difficult, as several data sources were used with significant missing data. This study included only surgical thyroid cancer patients, therefore some of the subtypes, such as anaplastic carcinoma, might be underrepresented.

## 6 CONCLUSION

This review describes the national landscape of thyroid cancer with its inherent challenges but affords the opportunity to work towards improving patient outcomes. This can be achieved at an individual patient level by standardizing diagnostics, reconsidering indications for surgery, and refining surgical technique. The impact can also be drawn nationally, which will require engagement with policymakers to improve access to radiological and pathological services. This study serves as an impetus toward a prospective national registry with real‐world data informing contextualized guidelines.

## AUTHOR CONTRIBUTIONS

**Wilhelmina Conradie**: Conceptualization, data curation, formal analysis, funding acquisition, investigation, methodology, project administration, resources, visualization, writing—original draft, writing—review & editing. **Thifhelimbilu Luvhengo**: Conceptualization, investigation, methodology, supervision, visualization, writing—review & editing. **Jeanne Adele Lübbe**: Conceptualization, investigation, methodology, supervision, visualization, writing—original draft, writing—review & editing. **Amir Afrogheh**: Conceptualization, data curation, methodology, project administration, resources, writing—review & editing. **Aneldi Bestbier**: Conceptualization, data curation, investigation, project administration, resources, writing—review & editing. **Mirza Bhuiyan**: Data curation, investigation, methodology, project administration, resources, writing—review & editing. **Ifongo Bombil**: Conceptualization, data curation, investigation, methodology, project administration, resources, writing—review & editing. **Sharon Raye Čačala**: Data curation, investigation, methodology, project administration, resources, writing—review & editing. **Lydia Cairncross**: Data curation, investigation, methodology, project administration, resources, supervision, writing—review & editing. **Chanel Changfoot**: Data curation, investigation, methodology, project administration, resources, writing—review & editing. **Jenny Edge**: Investigation, methodology, project administration, resources, writing—review & editing. **Brandon S. Jackson**: Data curation, investigation, methodology, project administration, resources, writing—review & editing. **Hansjörg S. Jehle**: Data curation, investigation, methodology, project administration, resources, supervision, writing—review & editing. **Lusanda Jonas**: Data curation, investigation, methodology, project administration, resources, writing—review & editing. **Mpoyi Ruphin Lukusa**: Data curation, investigation, methodology, project administration, resources, writing—review & editing. **Malose Makgoka**: Data curation, investigation, methodology, project administration, resources, writing—review & editing. **Lindi Martin**: Conceptualization, data curation, formal analysis, investigation, methodology, project administration, resources, validation, writing—review & editing. **Daniel Nel**: Data curation, investigation, methodology, project administration, resources, writing—review & editing. **Mohamed Quraish Patel**: Project administration, resources, writing—original draft, writing—review & editing. **Nosisa Thabile Sishuba**: Data curation, investigation, methodology, project administration, resources, writing—review & editing. **Rubina Razack**: Conceptualization, data curation, validation, writing—original draft, writing—review & editing. **Karin Baatjes**: Conceptualization, investigation, methodology, supervision, visualization, writing—original draft, writing—review & editing.

## CONFLICT OF INTEREST STATEMENT

The authors have no conflict of interest.

## ETHICS STATEMENT

This study was approved by the Health Research Ethics Committee of the University of Stellenbosch (S23/05/115). As this is a retrospective record review, a waiver of consent was obtained.

## CONSENT FOR PUBLICATION

All authors agree with the publication of this article.
